# Seasonal Spatial Distribution of *Metapenaeopsis provocatoria longirostris* in the Southern Yellow and East China Seas and Habitat Area Variation Prediction Under Climate Scenarios

**DOI:** 10.3390/biology14101328

**Published:** 2025-09-26

**Authors:** Min Xu, Yong Liu, Hui Zhang, Jianzhong Ling, Huiyu Li

**Affiliations:** Key Laboratory of Fisheries Remote Sensing Ministry of Agriculture and Rural Affairs, East China Sea Fisheries Research Institute, Chinese Academy of Fishery Sciences, Shanghai 200090, China; xuminwzy@aliyun.com (M.X.); liuy@ecsf.ac.cn (Y.L.); zhangh@ecsf.ac.cn (H.Z.)

**Keywords:** East Asia, stock assessment, total allowable catch, climate warming, migration, shrimp, Taiwan warm current, fisheries management

## Abstract

**Simple Summary:**

Crustaceans are currently facing important threats to their survival due to heavy fishing pressure and climate warming. At the same time, very limited ecological information is available for the shrimp species *Metapenaeopsis provocatoria longirostris* in the East China Seas. To identify them, we conducted field surveys from 2018 to 2019 and employed ensemble models under climate scenarios. We found that this species exhibited a decrease in biomass and abundance throughout the year, with values being higher in autumn than in winter, spring, and summer, in this order, as well as a decrease in size, with values being higher in spring than in summer and autumn/winter. With regard to its migration route, we argued that adults mainly spawn in spring in the fishing grounds of Mindong and Yuwai, preferring higher water temperature and salinity as well as greater depth. Recruitment occurs in summer in the inshore area near Yushan Island, which is used for feeding and as a nursery area, and in autumn, recruits migrate to the offshore Yuwai area. Then in winter, the main cohorts migrate southward to the fishing grounds of Wentai and Mindong for overwintering. Our findings provide key life history trait information for the resource management and plans of *M. provocatoria longirostris* in the future.

**Abstract:**

In real fisheries management practices, ecological information on the seasonal distribution patterns and characteristics of marine economic fauna and responses to climate change is necessary. In this study, we analyzed data obtained from surveys conducted between 2018 and 2019 in the southern Yellow and East China Seas, using ensemble models to predict variations in the habitat area of *Metapenaeopsis provocatoria longirostris* across seasons and under different climate scenarios. The highest abundances were observed at the following water temperature and salinity conditions, respectively: 18.5 °C and 34.5‰–35‰ in spring, 18.9–28 °C and 33.4–34.6‰ in summer, 18.6–21.7 °C and 33.5–34.4‰ in autumn, and 18–21.5 °C and 34‰ in winter. The major cohort was concentrated at depths of 100–110 m and 85–105 m in spring and summer, respectively, and at 80–100 m and 70–120 m in autumn and winter, respectively. In spring and winter, *M. provocatoria longirostris* was distributed in the continental shelf waters of the East China Sea, at wider salinity (30–35‰) and water temperature (8–26 °C) ranges, whereas in summer and autumn, the distribution shifted offshore. The values predicted for habitat loss under different climate scenarios were ranked as follows: ~70% loss under SSP585-2100 > ~50% loss under SSP370-2100 > ~30% loss under SSP245-2100 > ~15% loss under SSP126-2100 and SSP370-2050 > ~1–5% under SSP126-2050, SSP245-2050, and SSP585-2050. No great gains in habitat were predicted under any scenario. Our findings can contribute to the establishment of appropriate fisheries management schemes for the rational exploitation of *M. provocatoria longirostris*. Our predictions can assist in improving fisheries management practices within the context of climate change.

## 1. Introduction

Due to climate change, the Earth’s oceans are increasingly warm. Projections show that by the 2100s, the average ocean surface temperature will increase by 1.8–4 °C [1]. The average annual sea surface temperature in the Taiwan Strait has increased by more than 1 °C from 1957 to the present, with warming being more intense in winter (the highest average warming by 3.8 °C in February) [2]. Sea bottom salinity, which was relatively stable at 28.7‰ between the 1950s and the 1980s, reached 30‰ in the 2000s, increasing annually by 0.105‰ [3]. Climate change is known to contribute directly or indirectly to changes in the biomass, reproduction, recruitment, and distribution of marine biota, with invertebrates (including shrimps) being generally more sensitive than fishes [4].

At the same time, in Chinese waters, fishing pressure, in terms of fishing power (kw) and number of trawlers deployed, has increased more than twofold since the early 1990s [5]. The catch per unit effort (CPUE) of *Metapenaeopsis haanii* in the bottom trawl fishery decreased by 50% from the early 1990s to 2018–2019 in the southern Taiwan Strait [6]. Heavy exploitation has led to a decrease in both CPUE and shrimp size [4].

In marine crustaceans, climate warming (leading to warmer seawater) and intensive fishing pressure may reduce the size at maturity and cause earlier spawning [7]. For example, it has been shown that crabs (i.e., *Cancer irroratus* and *C. pagurus*) in warm waters mature earlier and spawn at smaller sizes than those in cool waters [8]. Similarly, higher seawater temperatures and smaller size at maturity were shown to result in an earlier spawning peak in *Charybdis natator* in the southern Taiwan Strait [4]. Heavy fishing pressure and climate warming pose important challenges to the management of crustacean species.

The genus Metapenaeopsis has a worldwide distribution and is important ecologically and commercially [9,10], but very few studies focus on their ecological and biological characteristics [11,12,13]. Rahman and Ohtomi (2018) found that the body weight of female velvet shrimp *Metapenaeopsis sibogae* was significantly larger than that of the males [13]. Tzeng et al. (2005) found that the instantaneous rate of natural mortality of *Metapenaeopsis barbata* was 2.69 year^−1^ in females [14]. Muralidharan et al. (2024) found that *Metapenaeopsis andamanensis* exhibited a rising trend from 2013 to 2020 [15].

The potential resource capacity of *Metapenaeopsis provocatoria longirostris* Crosnier (belonging to Penaeidae), previously known as *Metapenaeopsis philippinensis* and *Metapenaeopsis philippi*, is estimated at 10,000 to 20,000 tons in the East China Sea [16]. This species, along with *Parapenaeus fissuroides*, *Solenocera koelbeli*, *S. melantho*, *S. alticarinata*, and *Metapenaeopsis barbata*, has been a key commercial shrimp species for trawling fisheries operating in the southern and middle East China Sea since 1985 [16]. Mohale et al. (2025) found that the diet composition included digestive material (31%), detritus (29%), and copepods (20%) [17]. Muralidharan et al. (2023) found that the primary diet component of the species *Metapenaeopsis andamanensis* included detritus, forminifera, and crustaceans, followed by gastropods and fish [18]. Shrimps also play an important role in the structure and functioning of marine ecosystems, occupying intermediate to higher trophic levels in the food web and regulating productivity [19]. Thus, understanding the seasonal distribution of shrimp species under different climate scenarios is pivotal for resource conservation and fisheries management.

Currently, very limited ecological and biological information is available on *M. provocatoria longirostris*. Assessing the impact of climate change on its distribution and abundance can provide important data for developing management strategies for climate adaptation. Chandravanshi et al. (2024) argued that a comprehensive understanding of the seasonal–spatial distribution pattern and migration route is very important to design effective conservation programs [20].

In the present study, using data from surveys conducted between 2018 and 2019, we aimed to elucidate the effects of environmental variables (water temperature, salinity, and depth) on the seasonal distribution and characteristics of *M. provocatoria longirostris* (i.e., biomass, abundance, and individual size) in various fishing grounds in the southern Yellow and East China Seas and explored a potential migration route. We employed ensemble models to both determine spatial distribution characteristics and predict habitat patterns across seasons and under different scenarios, i.e., shared socioeconomic pathways (SSPs) SSP1-2.6, SSP2-4.5, SSP3-7.0, and SSP5-8.5. Our study can provide key initial information and baseline data for policy makers and fishermen that can assist in the conservation and sustainable management of *M. provocatoria longirostris* within the context of climate warming, as well as improving our knowledge of how marine shrimp species respond to climate change.

## 2. Materials and Methods

### 2.1. Geographic Characteristics of the Study Region and Survey Procedures

**Figure 1:**
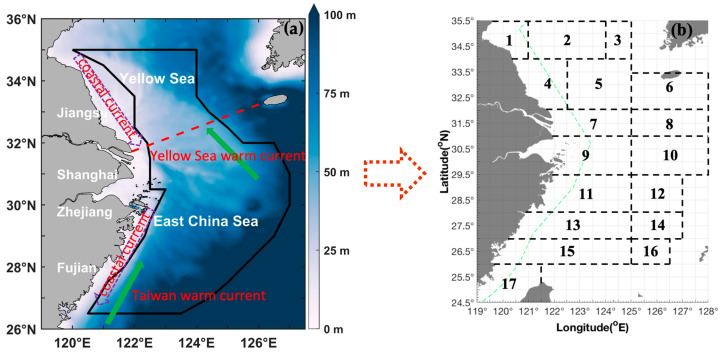
(**a**) Map of the study area (26.5–35° N, 120–127° E), which is denoted by a red dotted line in the East China Sea region and includes the southern Yellow and East China Seas. The red dashed line indicates the boundary between the Yellow Sea and the East China Sea. (**b**) The black boxes and numbers represent the following fishing grounds: (1) Haizhou Bay, (2) Lianqingshi, (3) Liandong, (4) Lvsi, (5) Dasha, (6) Shawai, (7) Yangtze River mouth, (8) Jiangwai, (9) Zhoushan, (10) Zhouwai, (11) Yushan, (12) Yuwai, (13) Wentai, (14) Wenwai, (15) Mindong, (16) Minwai, and (17) Minzhong. The green dashed line indicates line of closed fishing area for bottom trawl fishery by motorboat.

The main currents in the southern Yellow Sea and East China Sea include the China Coastal Current, the Yellow Sea Warm Current, and the Taiwan Warm Current (Figure 1). The China Coastal Current, which is the surface current in the study area, flows southward along the Chinese coast from Bohai Gulf to Taiwan (Figure 1). The Yellow Sea Warm Current, a branch of the Kuroshio Current, flows northward along the central axis of the Yellow Sea. This current is very shallow but strong and causes water in the central Yellow Sea to be several degrees warmer than that in the coastal regions (Figure 1). The Taiwan Warm Current, partly originating as an offshoot of the Kuroshio Current just northeast of Taiwan, carries warm water into the East China Sea (Figure 1). The Yangtze River, the third largest river in the world with an average annual discharge of 8,961,011 m^3^, can also influence surrounding currents as it enters the sea [21].

We performed bottom trawling surveys in the southern Yellow and East China Seas between 2018 and 2019 using a trawl net with a cod-end mesh of 20 mm, a height of 10–15 m, a headline of 72.24 m, and a groundline of 82.44 m [22]. The net was towed by fisheries research vessels in autumn (2–11 November 2018; 41,294.44 g∙h^−1^ [67.6%] of total catch per unit effort by weight [CPUE_w_] and 35,515.61 [72.8%] ind∙h^−1^ of total catch per unit effort by number [CPUE_n_]), winter (4–27 January 2019; 12,716.33 [20.8%] g∙h^–1^ of total CPUE_w_ and 9750.42 ind∙h^−1^ [20%] of total CPUE_n_), spring (22 April–10 May 2019: 4897.74 g∙h^−1^ [8%] of total CPUE_w_ and 2194.43 ind∙h^−1^ [4.5%] of total CPUE_n_), and summer (13 August–27 September 2019; 2212.31 g∙h^−1^ [3.6%] of total CPUE_w_ and 1313.82 ind∙h^−1^ [2.7%] of total CPUE_n_). The average trawl speed was 3 knots, and all tows were conducted for approximately 1 h at each station. In total, 519 valid tows were included in this study (127 conducted in autumn, 111 in winter, 141 in spring, and 140 in summer). The biomass and abundance of *M. provocatoria longirostris* in different seasons were ranked in the following order: autumn > winter > spring > summer. Growth for females and males was calculated as follows: W_♀_ = 0.1338 × 10^−4^ × (*L*)^2.8723^ and W_♂_ = 0.2839 × 10^−5^ × (*L*)^3.2496^ [16].

The catches were analyzed in the laboratory (East China Sea Fisheries Research Institute, Shanghai) to identify *M. provocatoria longirostris* and determine its occurrence at each station. The total sample for each station was counted and weighed to the nearest 0.10 g of wet weight, and the catch density of *M. provocatoria longirostris* was calculated as biomass density per unit of sampling time (g∙h^−1^) and density per unit of sampling time (ind∙h^−1^). The average individual weight (AIW) at each station was defined as CPUE_w_ divided by CPUE_n_. Environmental variables, including water depth, water temperature, and salinity, were measured at each station using a conductivity–temperature–depth profiler (SBE-19; SeaBird-Scientific, Bellevue, WA, USA) [23].

### 2.2. Ensemble Model and Climate Scenarios

Species distribution models are commonly used in ecology and biodiversity studies to predict the potential distribution of species [24]. In this study, ensemble models were employed to describe and predict the relationship between *M. provocatoria longirostris* and environmental variables. The following 10 methods were used to predict habitat distribution: artificial neural network (ANN), classification tree analysis (CTA), flexible discriminant analysis (FDA), generalized additive model (GAM), generalized boosting model (GBM), generalized linear model (GLM), multiple adaptive regression splines (MARS), random forest (RF), surface range envelope (SRE), and extreme gradient boosting training (XGBOOST). The validation, selection of environmental variables, and procedures of modeling SDM are detailed in Supplementary File S1.

Models were built using the “biomod2” package 4.2-6-2 (https://www.maths.bris.ac.uk/R/web/packages/biomod2/) (accessed on 18 September 2025) in the Biomod2 platform for ensemble species distribution modeling. A random 80:20 ratio split was applied for training and testing the data, respectively, to construct the algorithms for the 10 above-mentioned methods using random cross-validation [25]. Mean survey data over 4 months and single-season data were used to produce annual and seasonal models, respectively. All data used in the models were obtained from the surveys conducted in this study. Future climate data were obtained from the Coupled Model Intercomparison Project (Phase 6). Marine data layers for ecological modeling, including sea surface temperature (SST), sea bottom temperature (SBT), sea surface salinity (SSS), and sea bottom salinity (SBS), were obtained from the website Bio-ORACLE (https://bio-oracle.org/index.php, accessed on 15 August 2025). Four SSP scenarios, i.e., SSP1–2.6, SSP2–4.5, SSP3–7.0, and SSP5–8.5, were considered for mid-term forecasts in 2040–2050 (referred to as the 2050s) and long-term forecasts in 2090–2100 (the 2090s).

## 3. Results and Discussion

### 3.1. Seasonal Distribution and Characteristics of M. provocatoria longirostris

**Figure 2:**
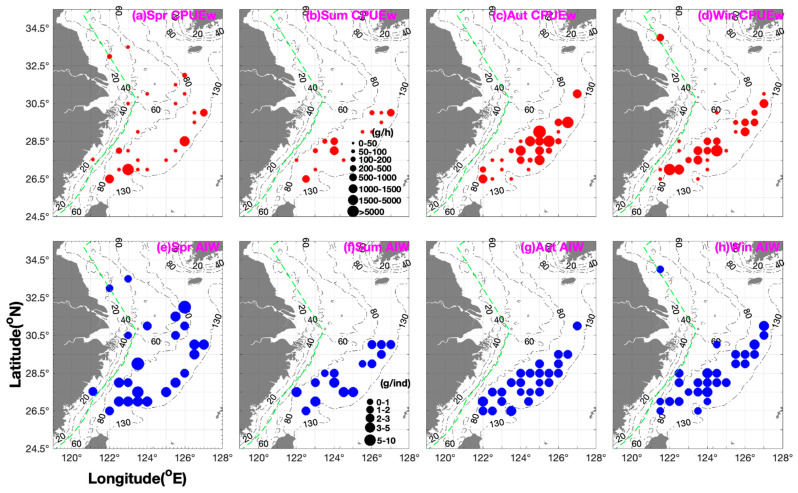
Seasonal spatial distribution patterns of *Metapenaeopsis provocatoria longirostris* catch per unit effort by weight (CPUE_w_; g·h^−1^) are shown in red (grouped into 0–50, 50–100, 100–200, 200–500, 500–1000, 1000–1500, 1500–5000, and >5000 g·h^−1^), and AIW (g·ind^−1^) data are shown in blue (grouped into 0–1, 1–2, 2–3, 3–5, and 5–10 g·ind^−1^). (**a**–**d**) CPUE_w_ in (**a**) spring, (**b**) summer, (**c**) autumn, (**d**) winter; (**e**–**h**) AIW in (**e**) spring, (**f**) summer, (**g**) autumn, and (**h**) winter.

The seasonal distribution of *M. provocatoria longirostris* and its characteristics in terms of biomass, number, and individual size in relation to environmental variables (i.e., SST, SBT, SSS, SBS, and depth) in various fishing grounds in the southern Yellow and East China Sea are summarized in Table 1 and Figure 2.

In spring, mean CPUE_w_ and CPUE_n_ were highest in the fishing grounds of Mindong (>50%) and Yuwai (>25%), followed by those in Zhouwai, and lowest in Dasha, Jiangwai, Zhoushan, and Wentai (Table 1). Mean AIW values were ranked in the following descending order: fishing grounds of Jiangwai (adult cohort) > Yuwai (adult cohort) > Wentai and Mindong (mixed cohorts of adults and juveniles) > Zhouwai (mixed cohorts) > Zhoushan (mixed cohorts) > Dasha (juvenile cohort) (Table 1 and Figure 2). As shown in Table 1, the adult cohort in Jiangwai and the juvenile cohort in Dasha preferred lower water temperature, with the latter still preferring slightly higher temperatures than the former. The two cohorts exhibited similar preferences for salinity (with juveniles thriving under a wider range of salinities compared to adults) and were observed in offshore and inshore areas, respectively. The ranking of CPUE_w_ and CPUE_n_ values in terms of longitudinal coordinates was 123° E–123.5° E and 126° E–127° E > 121.13° E–122.5° E > 124° E–125.5° E, and that of the AIW values was 126° E–127° E > 123° E–123.5° E > 124° E–125.5° E > 121.13° E–122.5° E (Table 1 and Figure 2).

In summer, mean CPUE_w_ and CPUE_n_ were highest in the fishing grounds of Yushan (30–40%), followed by those in Zhouwai, Wentai, and Mindong (~20%) and Yuwai (Table 1), indicating that recruits migrate in a scattered manner from the offshore area to the inshore area for feeding. Mean AIW values (most of them from recruits) were highest in the fishing grounds of Wentai and Mindong, followed by those in Zhouwai and Yuwai, and lowest in Yushan (Table 1). The ranking of CPUE_w_ and CPUE_n_ values in terms of longitudinal coordinates was 124° E–124.5° E > 122° E–122.5° E > 126° E–127° E > 123° E–123.5° E > 125° E–125.5° E and that of AIW values was 122° E–122.5° E and 124° E–124.5° E and 126° E–127° E > 123° E–123.5° E and 125° E–125.5° E (Table 1 and Figure 2).

In autumn, mean CPUE_w_ and CPUE_n_ were highest in the fishing grounds of Yuwai (~90%) and Wentai (~10%), followed by those in Mindong (Table 1), indicating that the recruits migrate to offshore areas for overwintering as water temperature decreases. In contrast, mean AIW values were higher in Mindong than in Yuwai and Wentai (Table 1). Correspondingly, most of the biomass was found at depths of 80–110 m, SSTs of 22–23.5 °C, SBTs of 18.5–21.8 °C, and SSS and SBS values of 33.5–34.5‰ (Table 1). The ranking of CPUE_w_ and CPUE_n_ values in terms of longitudinal coordinates was 125° E–125.5° E > 126° E–127° E > 124° E–124.5° E > 122° E–122.5° E > 123° E–123.5° E, showing an obvious increasing trend moving offshore, whereas the ranking of AIW was 122° E–122.5° E and 123° E–123.5° E > 126° E–127° E > 125° E–125.5° E > 124° E–124.5° E, showing that the adult and juvenile cohorts occupy the inshore and offshore areas, respectively (Table 1 and Figure 2).

In winter, the ranking of mean CPUE_w_ and CPUE_n_ values at fishing grounds was Wentai > Mindong > Lvsi, Yushan, and Yuwai > Zhouwai, and that of AIW values was Yushan > Zhouwai > Yuwai and Wentai > Lvsi and Mindong (Table 1 and Figure 2). Correspondingly, most of the biomass was located in waters with higher SBT (18–22 °C) and SSS and SBS of 34–35‰ (Table 1). In terms of longitudinal coordinates, the ranking of CPUE_w_ and CPUE_n_ values was 122° E–122.5° E > 124° E–124.5° E > 123° E–123.5° E and 126° E–126.5° E > 127° E > 121.5° E > 125° E–125.5° E and that of AIW values was 127° E > 122° E–122.5° E, 124° E–124.5° E, 125° E–125.5° E, and 126° E–126.5° E > 121.5° E and 123° E–123.5° E (Table 1 and Figure 2). These results show that in winter, the recruits migrate southward to warmer areas for overwintering, and in spring, most of them migrate to Mindong for spawning, with another spawning group migrating to the fishing grounds of Jiangwai and Yuwai. Choi et al. (2005) concluded that the species *Metapenaeopsis dalei* produced one cohort per year [26]. Chen et al. (2014) identified the species *Metapenaeopsis palmensis* as a multiple spawner [11]. Rahman and Ohtomi (2017) argued that the species *Metapenaeopsis sibogae* spawned throughout the year but peaked from September to October [12].

The ranking of mean and upper limit CPUE_w_ and CPUE_n_ values in single seasons was autumn > winter > spring > summer (Table 2), indicating that due to increasing fishing pressure, the population size in spring and summer was the smallest. Song et al. (2008) found an annual mean CPUE_w_ of 1230 g∙h^−1^ for *M. provocatoria longirostris*, with specific seasonal values ranked in the following order: summer (2140 g∙h^−1^) > spring (1390 g∙h^−1^) > autumn (720 g∙h^−1^) > winter (650 g∙h^−1^) [16].

The ranking of mean AIW values in single seasons was spring > summer > autumn and winter, whereas that of upper limit AIW values was spring > autumn and winter > summer, with the lower limit values ranging from 0.35 to 0.8 g∙ind^−1^ (Table 2). These results revealed that juveniles were present across the four seasons: after adults spawn in spring, juvenile recruitment occurs in summer, and recruits seek nursery areas for overwintering in autumn and winter.

Song et al. (2008) suggested that *M. provocatoria longirostris* mainly concentrated in the fishing grounds of Mindong and Wentai, with some groups being located in the fishing grounds of Yushan and Zhoushan [16]. Adults begin to breed in April, with a breeding peak in June and a period of May to July [16]. Recruits have a body length of 25–35 mm in May, and after August, the number of new recruits increases with accelerated growth speed [16]. The individuals increase in size between August and February of the following year (with body lengths of 37–48 mm in August, 40–58 mm in November, and 50–65 mm in February), becoming the main fishing target of beam shrimp trawl fisheries in spring and summer [16]. Between June and July, individuals grow up to 60 to 70 mm in length, and they gradually die after the spawning phase, their abundance decreasing after July [16]. After December, the cohort from the previous year disappears and is replaced by the new generation [16].

### 3.2. Seasonal Variations in Biomass and Abundance Under Varying Environmental Conditions

**Figure 3:**
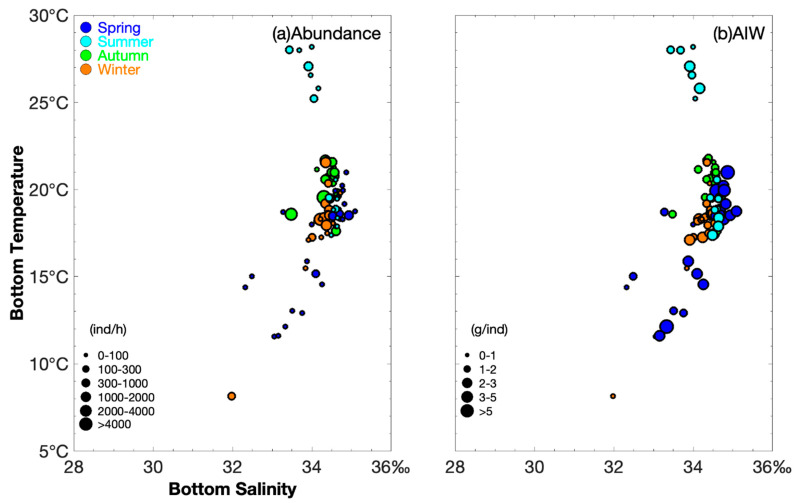
Relationship between bottom salinity (‰) and bottom temperature (°C) for catch per unit effort by number (CPUE_n_) size classified by group (0–100, 100–300, 300–1000, 1000–2000, 2000–4000, and >4000 ind·h^−1^) and average individual weight (AIW) size classified by group (0–1, 1–2, 2–3, 3–5, >5 g·ind^−1^). The data for spring, summer, autumn, and winter are denoted by solid blue, light blue, green, and brown circles, respectively. (**a**) Sea bottom temperature vs. sea bottom salinity for CPUE_n_; (**b**) sea bottom temperature vs. sea bottom salinity for AIW.

The abundance of *M. provocatoria longirostris* varied across seasons, with the highest values (corresponding to CPUE_n_) observed at the following SBTs and SBSs, respectively: spring, 18.5 °C and 34.5‰–35‰, CPUE_n_ > 200 ind∙h^−1^; summer, 18.9–28 °C and 33.4‰–34.6‰, CPUE_n_ > 100 ind∙h^−1^; autumn, 18.6–21.7 °C and SBS 33.5‰–34.4‰, CPUE_n_ > 100 ind∙h^–1^; and winter, 18–21.5 °C and SBS 34‰, CPUE_n_ > 1000 ind∙h^−1^ (Figure 3).

In spring, AIW values were >5 g∙ind^−1^ at SBTs and SBSs of 12.1–19.9 °C and 33.3‰–34.6‰, respectively, with a narrow salinity range for the spawning cohort, whereas AIW values were <1 g∙ind^−1^ at SBTs and SBSs of 11.6–14.4 °C and 32.3‰–33‰, indicating that juveniles prefer low-temperature and salt-fresh water (Figure 3). In summer, autumn, and winter, AIW values were <1 g∙ind^−1^ at SBT and SBS values of 25.2 °C and 34‰, 19.8–21.6 °C and 34.5‰, and 17.9–20.4 °C and 34.7‰, respectively (Figure 3).

In spring and summer (CPUE_w_ > 200 g∙h^−1^), shrimp concentrated at depths of 100–110 m and 85–105 m, whereas in autumn and winter (CPUE_w_ > 1000 g∙h^−1^), they concentrated at 80–100 m and 70–120 m (Figure 2 and Figure 3). Song et al. (2008) suggested that *M. provocatoria longirostris* are distributed in the East China Sea south of 30.5° N, mostly at depths of 80 to 100 m, with lower abundances at depths < 80 m and >120 m [16]. In this study, in the fishing grounds of Wentai and Mindong, the highest abundance was detected at a depth of approximately 100 m. The optimal depth for this species was determined to be 80–100 m.

In addition to environmental variables in the study area, the cohort had a large value range for water temperature (8–26 °C) and salinity (SSS 30‰–35‰ and SBS 32‰–35‰) in spring and winter, with a value range of 20 m to 150 m (Table 3). In summer, individuals were distributed in offshore areas with higher water temperature and salinity, whereas in autumn, they migrated to locations even farther offshore (Table 3). Overall, *M. provocatoria longirostris* are distributed in the continental shelf waters of the East China Sea in spring and winter and move to areas farther offshore in summer and autumn. Accordingly, the optimal water temperatures and salinities for this species were determined to be 13–21 °C and 34‰–34.5‰ in summer and 13–18 °C and 34‰–34.5‰ in winter, respectively (Table 3).

### 3.3. Prediction of Habitat Loss Under Different Climate Change Scenarios and Across Seasons

In terms of predicted loss of suitable habitat for *M. provocatoria longirostris*, the climate change scenarios examined were ranked in the following descending order: SSP585-2100 (~70% loss) > SSP370-2100 (~50% loss) > SSP245-2100 (~30% loss) > SSP126-2100 and SSP370-2050 (~15% loss) > SSP126-2050, SSP245-2050, and SSP585-2050 (~1–5%) (Table 4). No gains in habitat area were predicted under any of these scenarios (Table 4), suggesting that this shrimp species is sensitive to climate change.

**Figure 4:**
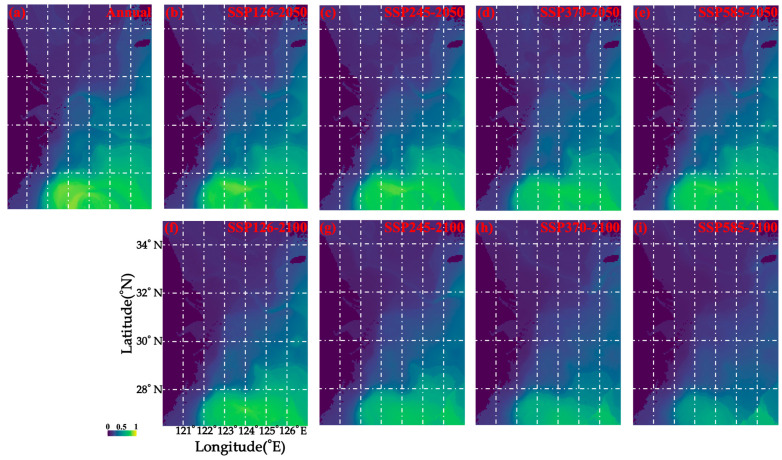
Predicted spatial habitat distribution patterns in the cases of (**a**) annual mean habitat, (**b**) SSP126 in 2050, (**c**) SSP245 in 2050, (**d**) SSP370 in 2050, (**e**) SSP585 in 2050, (**f**) SSP126 in 2100, (**g**) SSP245 in 2100, (**h**) SSP370 in 2100, and (**i**) SSP585 in 2100. The color of blue to green indicates the range from low to high suitability independently.

The areas with suitable habitats were located south of 28.5° N between 122 and 127° E, both based on the current data examined and under SSP126-2050, SSP126-2100, SSP245-2050, SSP245-2100, SSP370-2050, and SSP585-2050 (Figure 4). In the 2100s (under SSP370-2100 and SSP585-2100), the distribution of areas with suitable habitats is predicted to shrink to south of 27.5° N between 122 and 127° E (Figure 4).

**Figure 5:**
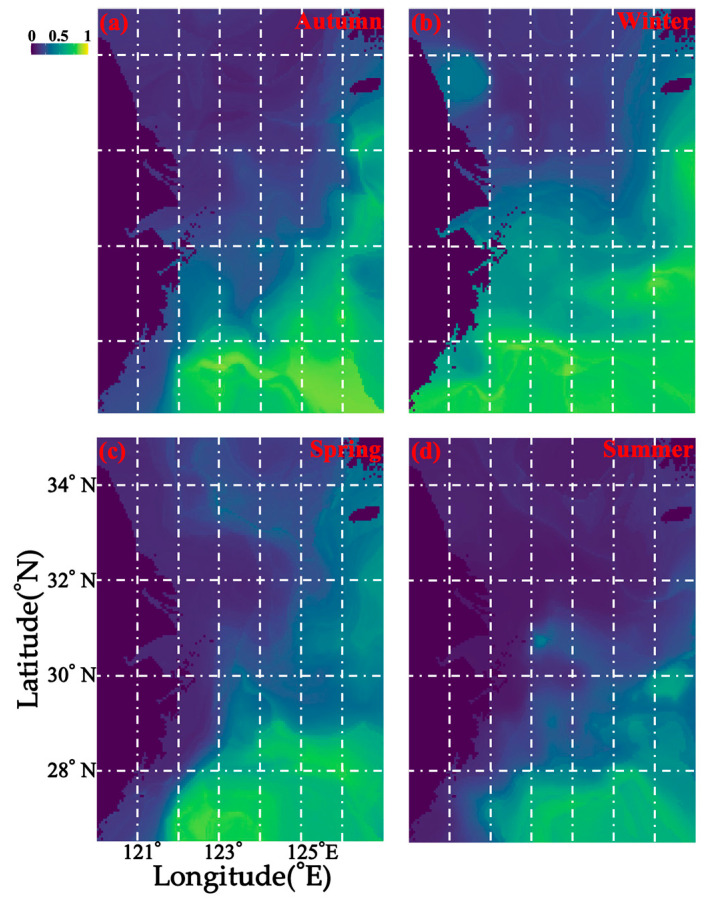
Current seasonal spatial predicted distribution patterns (**a**–**d**) from spring to winter in the study area based on data collected from 2018 to 2019. The color of blue to green indicates the range from low to high suitability independently.

With regard to the annual distribution of *M. provocatoria longirostris* in the East China Sea, individuals were shown to be concentrated south of 29° N between 121.5° E and 127° E in spring, and in summer, the habitat area further shrank to south of 27.5° N and between 122° E and 127° E (Figure 5). In autumn, individuals were distributed south of 29.5° N between 122° E and 127° E (except for the area extending from 28.5° N to 29.5° N and from 122° E to 125.5° E), with an area of 29.5° N–32.5° N 126° E–127° E (Figure 5). In winter, they were distributed south of 29.5° N between 120° E and 127° E (except for the area extending from 28.5° N to 29.5° N and from 122° E to 124° E), with an area of 29.5° N–32.5° N, 126.5° E–127° E (Figure 5).

### 3.4. Resource Conservation and Fisheries Management Strategies

Since the early 1980s, Chinese beam shrimp trawling fisheries have been operating in the northern (north of 30° N) and inshore areas of the southern East China Sea (at depths < 60 m) [16]. To manage and conserve fisheries resources in China’s exclusive economic zone, in 1995, the central government implemented a summer fishing moratorium on the fishing tools used in bottom trawling and sail sets, indirectly causing a dramatic increase in beam trawling for catching shrimp between July and August [16]. The use of illegal fishing methods such as electric stunning also exacerbated this situation [16]. Moreover, in China, most fisheries target multiple species, with ~90% of total marine catches being from non-selective trawlers, purse seines, gill nets, and set nets [5]. Fisheries targeting crustaceans are growing faster than other major fisheries [4]; therefore, more attention needs to be paid to their sustainable management.

Recently, some researchers started to question the effectiveness of minimum mesh size regulation from fisheries management and resource conservation perspectives [27,28,29], but Yang et al. (2021) suggested that increasing the mesh size in the diamond mesh cod-end of shrimp trawling is a simple option to mitigate the bycatch issue of undersized shrimp [30]. In 2015, to protect crustacean juveniles from overfishing, Zhejiang Province established a maximum allowable capture size and juvenile proportion in catches for economically important shrimp species [4]. In 2018, China’s central government established a minimum mesh size of 25 mm for the cod-ends of all trawls targeting shrimp species [4]. These provincial and national regulations also specified the minimum catch sizes and landing proportions for juveniles (http://hyyyj.fujian.gov.cn/xxgk/fgwj/201607/t20160721_1878278.htm; http://www.yyj.moa.gov.cn/tzgg/201404/t20140402_6300664.htm) (accessed on 18 September 2025) [4]. In addition to these measures, further conservation and fisheries management strategies might be required to mitigate the negative consequences of climate warming in China’s seas.

Here, we propose various measures to preserve *M. provocatoria longirostris* stocks. (1) Because the breeding season occurs from March to June and considering that China’s summer fishing moratorium begins on May 1st, it is necessary to implement a conservation plan for the adult cohort in March–April. A closed season to protect females bearing eggs can help the recovery of the recruitment and spawning stocks to achieve the maximum sustainable yield and meet both biological and economic objectives. (2) In the study area, fishing shrimp via beam trawling is allowed from August 1st, and we suggest postponing this starting date to after September 16 for *M. provocatoria longirostris* so as to protect recruitment. Given that this is a key prey species for various predators, such postponement can also benefit the recruitment of other economically important fishes. (3) Finally, considering the expected climate warming in the future, it is important to continue monitoring the resource status of *M. provocatoria longirostris* and establish a possible refuge area for the adult and recruit populations.

Our findings provide baseline data for the formulation of fishery management policies, suggesting modifications in the time and location of fishing. Within the context of climate change, it is important to protect new habitat areas, monitor habitat loss, and moderately utilize resources in stable habitats.

## 4. Conclusions

We found that, in spring (May), most adults migrate to the fishing grounds of Mindong and Yuwai for spawning, with some potentially spawning in Jiangwai, which are areas characterized by high salinity, high water temperature, and deep waters. The adult cohort spawns offshore, and then newborn juveniles migrate in a scattered manner to warmer waters inshore for feeding. In summer, the majority of recruits concentrate in the fishing ground of Yushan (nursery area), with some being distributed scatteredly in Zhouwai, Wenwai, and Mindong. In this season, most of the juveniles move to the inshore areas for feeding. In autumn, most of the recruits migrate from Yushan (inshore) to Yuwai (offshore), with some moving to the fishing ground of Wentai. In winter, the main cohorts migrate southward to the fishing grounds of Wentai and Mindong for overwintering. Our findings provide key information for regular stock assessment and management of *M. provocatoria longirostris* in the Yellow and East China Seas. In the future, an adaptive fisheries management strategy should be adopted by gathering seasonal distribution data, setting management goals, monitoring and assessing the effectiveness of implemented measures, and then reviewing what worked and what failed.

## Figures and Tables

**Table 1:** Mean and total values of CPUE_w_ (B), CPUE_w_% (B%), CPUE_n_ (N), CPUE_n_ (N%), AIW, and AIW% at different environmental conditions, i.e., sea bottom temperature (SBT), sea bottom salinity (SBS), and depth, in various fishing grounds in the southern Yellow and East China Seas across seasons.

	Mean Value					Total Value						Environmental Variable		
	B	B%	N	N%	AIW	B	B%	N	N%	AIW	AIW%	SBT (°C)	SBS (‰)	Depth (m)
Spring														
(5)	36.6	3.3%	47.3	8.9%	0.7	73.2	1.5%	94.7	4.3%	1.5	0.8%	11.6–14.4	32.3–33.1	20–45
(8)	37.2	3.4%	5.4	1%	5.5	74.5	1.5%	10.8	0.5%	11	6%	11.6–12.1	33.2–33.3	55–81
(9)	16.1	1.4%	10	1.9%	1.4	32.2	0.7%	20	0.9%	2.7	1.5%	15–18	32.5–34	41–49
(10)	98.8	8.9%	45.5	8.6%	2	395.2	8.1%	182.1	8.3%	7.8	4.3%	12.9–15.2	33.5–34.3	65–97
(12)	404.3	36.4%	218	41%	4.2	1213	24.8%	653.9	29.8%	12.7	6.9%	15.9–19.9	33.9–34.6	74–107
(13)	41.6	3.7%	19.8	3.7%	2.4	249.6	5.1%	119	5.4%	14.2	7.7%	18.6–20.2	33.3–34.8	40–104
(15)	476.7	42.9%	185.7	34.9%	2.5	2860	58.4%	1114	50.8%	134.2	72.9%	18.3–21	34.7–35.1	98–140
Summer														
(10)	177	23.7%	98	20.4%	1.7	531.1	24%	294.1	22.4%	5.2	19.9%	18.9–20.6	34.6	77–97
(11)	242.4	32.4%	201.8	42.1%	1.1	484.7	21.9%	403.6	30.7%	2.1	8.1%	25.2–28	33.4–34.1	70–84
(12)	14.8	2%	7.8	1.6%	1.6	44.4	2%	23.4	1.8%	4.8	18%	18.8–28.2	33.7–34.5	10–117
(13)	175	23.4%	82.7	17.2%	2.1	875.2	39.6%	413.7	31.5%	10.3	39.1%	17.9–27.1	33.9–34.6	75–101
(15)	138.5	18.5%	89.5	18.7%	2	276.9	12.5%	179	13.6%	3.9	14.9%	17.4–19.5	34.4–34.5	104–117
Autumn														
(12)	3665.1	86.5%	3161.6	86.9%	1.4	36,650.8	88.8%	31,615.6	89%	13.5	38%	18.5–21.7	33.5–34.6	82–107
(13)	406.1	9.6%	342.7	9.4%	1.2	3655.2	8.9%	3084	8.7%	10.6	29.8%	18.5–21.8	34.4–34.6	83–105
(15)	164.7	3.9%	136	3.7%	1.9	988.4	2.4%	816	2.3%	11.4	32.2%	17.5–19.9	34.5–34.7	85–135
Winter														
(4)	299.2	12.5%	300	16.4%	1							8.1	32	15
(10)	186	7.8%	123.9	6.8%	1.7	744.2	6%	495.7	5.2%	7	18.1%	15.5–19.2	33.8–34.3	55–100
(11)	245.3	10.3%	118.7	6.5%	1.9	735.8	5.9%	356.2	3.8%	5.8	15.2%	17.1–17.3	33.9–34	64–92
(12)	303.5	12.7%	219	12%	1.6	1517.3	12.2%	1095.2	11.6%	7.8	20.2%	17.5–18.8	34.4–34.6	88–114
(13)	652.7	27.4%	563.9	30.9%	1.5	5221.8	42.1%	4511.3	47.7%	12.1	31.5%	18.2–20.4	34.1–34.5	80–107
(15)	699.7	29.3%	498.7	27.3%	1	4198	33.8%	2992	31.7%	5.7	15%	17.9–21.6	34.2–34.7	69–145

Notes: fishing grounds of (4) Lvsi, (5) Dasha, (8) Jiangwai, (9) Zhoushan, (10) Zhouwai, (11) Yushan, (12) Yuwai, (13) Wentai, and (15) Mindong.

**Table 2:** Seasonal data for the mean value and value range of CPUE_w_ (unit: g·h^−1^), CPUE_n_ (unit: ind·h^−1^), and AIW (unit: g·ind^−1^) in autumn 2018 to summer 2019.

Factor	Spring	Summer	Autumn	Winter
Mean CPUEw at collection stations (g·h^−1^)	195.91	147.49	1651.78	470.98
Value range of CPUEw (g·h^−1^)	2–2012	1.4–693.6	2.8–27,686.26	2.8–3099.43
Mean CPUEn at collection stations (ind·h^−1^)	87.78	87.59	1420.62	361.13
Value range of CPUEn (ind·h^−1^)	0.92–788	1–324	1–24,575.88	4–2674.29
Mean AIW (g·ind^−1^)	2.6	1.76	1.42	1.46
Value range of AIW (g·ind^−1^)	0.7–8.7	0.8–2.4	0.7–2.8	0.35–2.75

**Table 3:** Seasonal data ranges of environmental factors (SST, SSS, SBT, SBS, depth) across seasons.

Factor	Spring	Summer	Autumn	Winter
SST (°C)	13.6–26	26.1–29	21.9–26.3	8.1–22.3
SSS (‰)	30.5–34.6	31.7–34.1	33.3–34.4	31.9–34.5
SBT (°C)	11.6–21	17.4–28.2	17.5–21.8	8.1–21.6
SBS (‰)	32.3–35.1	33.4–34.6	33.5–34.7	32–34.7
Depth (m)	20–140	10–117	82–135	15–145

**Table 4:** Percentages of habitat loss, gain, and overall habitat (gain minus loss) under various climate scenarios (SSP126-2050, SSP126-2100, SSP245-2050, SSP245-2100, SSP370-2050, SSP370-2100, SSP585-2050, and SSP585-2100).

Case	Loss%	Gain%	Gain% − Loss%
SSP126–2050	−1.67%	0.37%	−1.3%
SSP126–2100	−13.84%	0%	13.84%
SSP245–2050	−3.02%	0.12%	−2.91%
SSP245–2100	−30.15%	0%	−30.15%
SSP370–2050	−14.44%	0%	−14.45%
SSP370–2100	−54.41%	0%	−54.41%
SSP585–2050	−3.16%	0%	−3.16%
SSP585–2100	−72.34%	0%	−72.34%

## Data Availability

The original contributions presented in this study are included in the article. Further inquiries can be directed to the corresponding authors.

## Supplementary Materials

The following supporting information can be downloaded at https://www.mdpi.com/article/10.3390/biology14101328/s1, Supplementary File S1. Reference [31] is included in supplementary materials.
