# Supplementary material for: Seasonal Spatial Distribution of Metapenaeopsis provocatoria longirostris in the Southern Yellow and East China Seas and Habitat Area Variation Prediction Under Climate Scenarios
Source: Biology (Basel). 2025 Sep 26;14(10):1328. doi: 10.3390/biology14101328 (PMC12561830; doi:10.3390/biology14101328)
Supplement: Supplementary file 1 [file biology-14-01328-s001.zip › biology-3850201-supplementary.pdf]

Supplementary file S1

Independent scientific bottom trawling surveys were conducted in the southern Yellow and East China Seas (also called as East China Sea region) during 2018 and 2019. The surveys used a trawl net with a cod end mesh size of 20 mm and a height of 10–15 m that was towed by fisheries research vessels (the Zhongkeyu 211 and 212) in autumn, winter, spring, and summer. The study area covered 26.50–35.00° N, 120.00–127.00° E. The survey stations were determined using a sampling grid with dimensions of 30 min of latitude and 30 min of longitude (30′× 30′). The average trawl speed was 3 knots and all tows were conducted for approximately 1 h at each station. In total, 519 valid tows were included in this study: 127 stations in autumn, 111 stations in winter, 141 stations in spring, and 140 stations in summer.

The catches were analyzed in the laboratory to identify the species caught and assess their occurrence at each station. The total sample of the species in each station was counted and weighed to the nearest 0.10 g of wet weight, and the catch density of this species was calculated as biomass density per unit of sampling time ($g \cdot h^{-1}$) and individual numerical density per unit of sampling time ($ind \cdot h^{-1}$). The average individual weight (AIW) was defined as the $CPUE_w$ divided by the $CPUE_n$ at each station. Environmental variables, including water depth, water temperature, and salinity, were measured at each station using a conductivity–temperature–depth profiler (SBE-19; SeaBird-Scientific, Bellevue, WA, USA). SST (sea surface temperature) and SSS (sea surface salinity) were measured at 3 m below the surface, and SBT (sea bottom temperature) and SBS (sea bottom salinity) were measured 2 m above the sea bottom at sea depths < 50 m) and at 2–4 m above the bottom at sea depths > 50 m.

In this study, we used the species distribution model to describe and forecast the relationship between the species and environmental variables. SDM has been widely applied to forecasting the habitat distribution of marine animals in China's Seas and other sea areas. We used the following 10 algorithms to predict the habitat distribution of the species: artificial neural network (ANN), classification tree analysis (CTA), flexible discriminant analysis (FDA), generalized additive model (GAM), generalized boosting model (GBM), generalized linear model (GLM), multiple adaptive regression splines (MARS), random forest (RF), surface range envelope (SRE), and extreme gradient boosting training (XGBOOST). Araujo and New (2007) reported that the ensemble model that combines these 10 models was more advantageous compared with single models, and this can largely improve the robustness of the prediction and decrease analysis bias, thereby yielding more confidence in the predictions.

We used the "biomod2" package in the ensemble SDM planform. To run the model, the data set was separated into categories of 0 (absence) and 1 (presence), and an 80%:20% split was then randomly applied for training and testing data independently to construct the 10 algorithms using the random cross-validation method. Each algorithm was run 20 times to obtain 200 models to obtain stable results. We used the mean survey data over four months to produce the annual model, and used different seasonal data to produce the seasonal models. All the data used in the models were obtained from surveys conducted as part of this study. The performance of each algorithm was assessed by the index of the area under the receiver

operating characteristic curve (ROC) and the true skill statistic (TSS). Among these 200 models, we selected those that performed best (a threshold value of the receiver operating characteristic curve (AUC) > 0.8) and combined them into an ensemble model using the weighted average method. Details regarding the function and usage of variable importance can be found at (biomodhub.github.io/biomod2/reference/bm_VariablesImportance.html).

Future climate data were obtained from the Coupled Model Intercomparison Project Phase 6 (CMIP6), and environmental data such as SST, SBT, SSS, and SBS, were obtained from the website Bio-ORACLE: marine data layers for ecological modeling (https://bio-oracle.org/index.php). The four Shared Socioeconomic Pathways (SSPs) scenarios (SSP1–2.6, SSP2–4.5, SSP3–7.0, and SSP5–8.5) for 2040–2050 (the 2050s) and 2090–2100 (the 2090s) were used in this study. The SSP1–2.6 scenario is a sustainable development situation that emphasizes sustainability, low resource consumption, and low carbon emissions. The SSP2–4.5 scenario is the intermediate challenges scenario, with a radiative stabilization rate of 4.5 W m^{-2} beyond 2100. The SSP3–7.0 scenario contains a medium to high forcing regional rivalry pathway with a radiative stabilization rate of 7.0 W m^{-2}. Finally, the SSP5–8.5 scenario encompasses a fossil fuel-driven development situation characterized by high carbon emissions and assumes that future societies will rely heavily on fossil fuels to power economic growth.

Bias corrections were performed for SST, SSS, SBT, and SBS. Climate models, while foundational, possess intrinsic limitations that can introduce biases in projected environmental variables. These biases have the potential to compromise the precision of species distribution models. Bias correction of climate model raw data is essential to enhance the credibility of habitat distributions under future climate scenarios. The delta method is a prevalent technique in fisheries habitat prediction that effectively mitigates such biases. We employed this approach to calculate climate differences between contemporary and future datasets by applying corrections to raw data. Specifically, the delta method leverages discrepancies between observed and simulated baseline conditions to adjust simulations for time (*t*) periods (2040–2050 and 2090–2100).

Bias correction for time *t* in geographical location x was conducted as follows:

$$D_{\mathrm{sim}}^{\mathrm{DM}}(x,t) = D_{\mathrm{emp}}(x,0) + \left(D_{\mathrm{sim}}^{\mathrm{raw}}(x,t) - D_{\mathrm{sim}}^{\mathrm{raw}}(x,0)\right)$$

$$= D_{\mathrm{sim}}^{\mathrm{raw}}(x,t) + \left(D_{\mathrm{emp}}(x,0) - D_{\mathrm{sim}}^{\mathrm{raw}}(x,0)\right)$$

where $D_{\mathrm{emp}}(x,0) - D_{\mathrm{sim}}^{\mathrm{raw}}(x,0)$ represents the bias as the anomaly between observed and simulated environmental data at location x, and $D_{\mathrm{sim}}^{\mathrm{DM}}(x,t)$ denotes the bias-corrected temperature forecasts that were calculated by adding the bias to the simulated environmental data for time *t* in geographical location x.

Reference

Araujo, M.; New, M. Ensemble forecasting of species distributions. Trends. Ecol. Evol. 2007, 22, 42–47.
